# Supplementary material for: The Goldbeter-Koshland Switch in the First-Order Region and Its Response to Dynamic Disorder
Source: PLoS One. 2008 May 14;3(5):e2140. doi: 10.1371/journal.pone.0002140 (PMC2374878; doi:10.1371/journal.pone.0002140)
Supplement: Appendix S1 — (0.11 MB DOC) [file pone.0002140.s001.doc]

# Appendix S1

Here we show that case a and a2 cannot generate sigmoidal behavior, because the second derivative of as function of or is always monotonic.

## Case a

We examined the steady-state solution of Eq. (2) and (3) in the main text. First other quantities can be expressed by,

Then the physically meaningful solution of is

where,

Then,

The second derivative of to is monotonic, and approaches zero only when. Therefore, SR curve is hyperbolic and shows no sigmoidal behavior.

Next we examine the function dependence of on, the concentration of free S molecules. The steady state solution of has the form,

One can easily show that its second derivative to is mono-signed. Therefore, case a does not have a sigmoidal behavior whether the total concentration or free concentration of S is controlled as the signal.

## Case a2

The governing equations of this case (Figure 1a) read

with concentration constraints

In this case, an additional intermediate step is added upon case a. After some tedious but straightforward derivation, one obtains

with

Based on these equations it can be proved mathematically that the second derivatives of to and are both mono-signed. Therefore, case a2 gives no sigmoidal behavior.
